# Atrial Septal Defects Accelerate Pulmonary Hypertension Diagnoses in Premature Infants

**DOI:** 10.3389/fped.2018.00342

**Published:** 2018-11-23

**Authors:** Shilpa Vyas-Read, Lokesh Guglani, Prabhu Shankar, Curtis Travers, Usama Kanaan

**Affiliations:** ^1^Department of Pediatrics, Emory University, Atlanta, GA, United States; ^2^Department of Public Health Sciences, University of California, Davis, Davis, CA, United States; ^3^Biostatistics Core, Pediatric Research Alliance, Atlanta, GA, United States; ^4^Sibley Heart Center, Atlanta, GA, United States

**Keywords:** prematurity, atrial septal defect, pulmonary hypertension, echocardiogram, left-to-right shunt, neonate

## Abstract

Between 4 and 16% of extremely premature infants have late pulmonary hypertension (PH) (onset >30 days of life), and infants with PH have a higher risk of tracheostomy and death. Atrial septal defects (ASD) increase pulmonary blood flow and may promote PH in at-risk infants. The objective of this study was to determine if infants with ASD develop PH sooner than those without ASD. Infants who were born at < 32 weeks' gestation, with an echocardiogram on day of life > 30, and without congenital anomalies were included. Infants with and without ASD were evaluated for the time to PH diagnosis, defined as the day of the first echocardiogram that showed PH. A multivariable model with ASD and significant variables on PH and a Cox proportional hazard model evaluating time to PH was determined. Of the 334 infants with echocardiograms, 57 had an ASD and 26% of these developed PH vs. 12% without ASD (*p* = 0.006). Infants with PH had lower gestational age (25.2 vs. 26.2 weeks, *p* = 0.005), smaller birthweight (699 vs. 816 gm, *p* = 0.001), and more prematurity complications than infants without PH. More PH infants had maternal African-American race (63.9 vs. 36.1%), right ventricular dysfunction (23.9 vs. 3.2%, *p* < 0.001), right ventricular dilation (52.1 vs. 8.6%, *p* < 0.001), or right ventricular hypertrophy (51.2 vs. 10.1%, *p* < 0.001), than infants without PH. At 150 days of life, 78.1% (95% CI 64.6–86.9%) of infants with ASD survived without PH, compared with 90.9% (95% CI 86.7–93.8%) of infants without ASD, and the unadjusted hazard for development of PH for infants with ASD was 2.37 (95% CI 1.29–4.36). When significant clinical variables were controlled, infants with ASD had a 2.44-fold (95% CI 1.27–4.68) increase in PH, compared with infants without ASD. Most PH in infants with or without ASD was diagnosed by day of life 150, but infants with ASD had an over 2-fold increased hazard for PH during their neonatal hospitalization. Premature infants with ASD should be followed closely for PH development and further studies to investigate the optimal timing of closure are needed.

## Introduction

Late pulmonary hypertension (PH), or pulmonary hypertension beyond the first month of life, affects between 4 and 16% of extremely low birthweight infants (1–4). A diagnosis of PH, particularly when combined with a diagnosis of bronchopulmonary dysplasia (BPD), confers a 3.8-fold increased risk for tracheostomy, and up to half of infants experience mortality within the first few years of life (5–7). The gravity of these outcomes suggest a need for earlier identification and improved therapeutic approaches for at-risk premature neonates.

Although the accurate prediction of the risk for developing PH for individual patients is challenging, certain prenatal and neonatal diagnoses, such as intrauterine growth restriction, BPD, postnatal sepsis or necrotizing enterocolitis, can increase an infant's likelihood of being affected (8–15). In addition to prenatal and hospital factors, prospective investigations have found that there may be an enhanced risk for PH in premature infants with left-to-right shunts, particularly atrial septal defects (ASD) (16–18). Limited studies suggest that infants who have ASD have a much higher odds of developing PH, even when other markers of acuity such as gestational age and birthweight are controlled (14). Possible explanations of how left-to-right shunts, such as ASD, may contribute to pulmonary vasculature changes and PH have been postulated.

Infants with BPD have been shown to have decreased vascular growth, increased vascular tone and altered vasoreactivity secondary to multifactorial etiologies associated with preterm birth (19, 20). Potentially, the addition of a second insult, such as pulmonary overcirculation from anatomic shunts such as ASD, could further exacerbate vascular pathology. Animal models of pulmonary over circulation, in which an arterial-to-venous shunt is artificially created, have demonstrated increases in mean pulmonary artery pressure and in medial thickness of pulmonary arteries, similar to that found on the histology of the lungs of premature infants who have died with PH (21, 22). Further, animals models of flow-induced PH have increased pulmonary vascular reactivity and impaired endothelial-dependent, suggesting not only abnormalities in the structure but also in the function of the vasculature in the setting of prolonged excessive flow (16). Retrospective studies of infants born before 32 weeks' gestation have demonstrated that a higher proportion of infants with PH have ASD, than infants without PH (23). Although small left-to-right shunts will often close spontaneously after birth, larger and persistent ASDs may ultimately require interventional closure or surgery (24, 25). Small studies that have evaluated percutaneous transcatheter device closure of ASD in sick neonates have shown some improvements in respiratory status and medication use post-procedure, suggesting that, in some patients, treatment may improve outcomes (17, 18, 26). However, whether infants with ASD develop PH in the same time frame, or develop PH sooner than infants without ASD is unclear. A better understanding of the temporality of PH diagnosis in infants with atrial shunts may be used to guide timely diagnosis and treatment.

The main objective of this study was to determine if infants with ASD are more likely to develop PH within the first 250 days of life than infants without ASD. As secondary objectives, we explored clinical factors associated with ASD and with PH in a cohort of infants born at less than 32 weeks' gestation at birth admitted to a referral neonatal intensive care unit.

## Materials and methods

**Figure 1:**
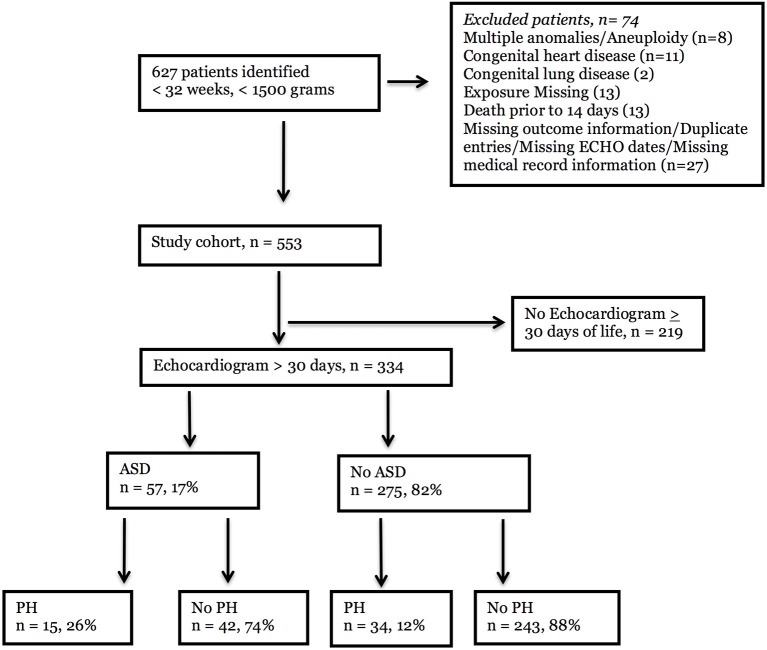
Flowchart of patient selection. An electronic health record database query was performed for < 32 weeks' gestation at birth, < 1,500 g birthweight, neonatal intensive care unit, and echocardiographic procedure. Six hundred and twenty-seven infants were identified, and 74 infants were excluded due to congenital anomalies or missing information, Of the 553 eligible infants, 334 had an echocardiogram after 30 days of life and could be evaluated for late pulmunory hypertension (PH). Of these, 57 infants had atrial septal defects (ASD) and 275 infants did not. PH was detected in 15 (26%) patients with ASD and 34 (12%) patients without ASD.

This study was carried out in accordance with the Human Research Protection Program of the Institutional Review Board at Emory University and at the Children's Healthcare of Atlanta. The protocol was approved by Emory University and Children's Healthcare of Atlanta. Written consent from human subjects was not obtained because the presented research presented no more than minimal risk of harm to subjects and the research involved no procedures for which written consent is normally required outside of the research context. The study population was a retrospective, observational cohort of patients at two quaternary pediatric referral centers within the Children's Healthcare of Atlanta (Egleston and Scottish Rite campuses) from January 2010 to September 2014, described previously (11). Infants who were less than 32 weeks' gestational age at birth, had a birth weight of less than 1,500 grams, were in the neonatal intensive care unit, and had an echocardiogram were identified using ICD-9 codes and were included. Patients were excluded if medical records were missing or if they had multiple anomalies/aneuploidy, congenital heart disease (other than atrial septal defect, ventricular septal defect, or patent ductus arteriosus), or congenital lung disease (see Figure 1). Infants who had an echocardiogram beyond 30 days of life were included in the final study cohort, to capture those infants with PH beyond the period of persistent pulmonary hypertension of the newborn or transitional physiology.

### Exposure and outcome

The primary exposure was the presence of an atrial septal defect (ASD) on any echocardiogram after day of life 30. Infants were labeled as having an “ASD” if they had a diagnosis of ASD or “ASD vs. patent foramen ovale (PFO)” as determined by the pediatric cardiologist reading the study. If infants had no atrial level communication, or they had only a PFO designated on the echocardiogram, they were labeled as having “No ASD.” Clinically, at our center, an ASD is generally defined as an atrial communication greater than 3 mm in diameter with clear margins and no significant tissue in the region of the defect, and a PFO is considered to be a smaller defect, less than 3 mm, with a flap of tissue in the mouth of the defect that restricts flow. The designation of “PFO vs. ASD” is used when the size of the defect is larger than the typical small PFO (often in the 3–5 mm range) but there are anatomic features such as a foraminal flap suggestive of a PFO. Often when the term “PFO vs. ASD” is used at our site, there is no restriction to the atrial level shunt.

The primary outcome was late pulmonary hypertension (late PH) on the first echocardiogram after 30 days. Pulmonary hypertension (PH) was defined as an echocardiogram that showed: (1) a patent ductus arteriosus (PDA) with bidirectional or right-to-left shunting; (2) a tricuspid regurgitation jet gradient of > 32 mm Hg with septal flattening, right ventricular hypertrophy, or right ventricular dilation; or (3) a tricuspid regurgitation jet velocity of > 45 mmHg. Echocardiograms were ordered at the discretion of the attending neonatologist and interpreted by pediatric cardiologists.

### Clinical variables

The following variables were abstracted from the infant medical record: (1) maternal drug use, the use of tobacco and/or alcohol; (2) illicit drug use, including the use of illegal drugs such as cannabis, amphetamines, or other substances; (3) infant race and gender; and (4) prenatal and intrapartum complications. The following discrete variables were abstracted from the clinical data warehouse using ICD-9 codes: (1) intraventricular hemorrhage; (2) necrotizing enterocolitis; (3) retinopathy of prematurity; (4) medication use; (5) respiratory support; and (6) positive culture results in the hospital microbiology laboratory. Death was defined as mortality from any cause during the hospital course.

### Echocardiographic variables

The echocardiographic characteristics of infants in the cohort were determined by the echocardiogram that first showed PH for infants in the PH group, or by the final echocardiogram of the neonatal hospitalization for the No PH group. Directionality of the shunt through an ASD, ventricular septal defect, or patent ductus arteriosus (PDA) was designated as (1) left-to-right (2) bidirectional or (3) right-to-left, and the PDA size was graded as (1) none, tiny, or small or (2) moderate or large by the pediatric cardiologist at the time of the interpretation of the echocardiogram. The tricuspid regurgitation jet velocity (TRJV) was graded as (1) normal, < 32 mmHg (2) mildly elevated, 32–44 mmHg (3) moderately elevated 45–60 mmHg, and (4) severely elevated > 60 mmHg at that time. Septal flattening was defined subjectively into “none or mild” or “moderate or severe”categories by the pediatric cardiologist interpreting the echocardiogram. Right ventricular dilatation, hypertrophy, and dysfunction were defined as either present or absent. Ventricular septal defects were defined as (1) intact, tiny, and small or (2) moderate, large, or multiple. Systolic blood pressure and diastolic blood pressure were recorded at the time of the echocardiogram showing PH or the final echocardiogram of the neonatal stay (for the No PH group).

### Descriptive statistics

Two-sample *t*-tests for normally distributed variables, and Wilcoxon rank sum tests for skewed distributions were utilized. For categorical variables, chi-square tests of proportion were used to compare outcome groups unless the cell frequency was < 5, in which case the Fisher's exact test was used.

### Univariable and multivariable analyses

Following descriptive analyses, a multivariable model was developed to evaluate the effect of ASD on the time to echocardiographic PH diagnosis. After determining that the proportional hazard assumption holds, significant co-variates from the descriptive analyses were entered into a Cox proportional hazard model with birth weight, intrauterine growth restriction, respiratory support at 28 days and maternal race forced into the model. Using backward elimination, variables that no longer met statistical significance were removed from the model leaving a final model that included ASD, respiratory support at 28 days, sepsis, African-American maternal race, birth weight and intrauterine growth restriction. Infants were censored from the analyses at the time of discharge from the neonatal intensive care unit, PH diagnosis, or death. All statistical procedures were performed using SAS 9.4 statistical software and the level of significance for comparisons was a *p*-value < 0.05.

## Results

The number of infants that met the criteria of being less than 32 weeks' gestation, of weighing less than 1,500 grams at birth, of being in a neonatal intensive care unit, and of having had an echocardiogram was 627 (Figure 1). Of these, 74 infants were excluded due to congenital anomalies, congenital heart or lung disease, death in the first 14 days of life, or missing exposure, outcome, or echocardiography information. Of the 553 eligible participants, 334 infants who had an echocardiogram beyond 30 days of life were included in the final study cohort. Infants who had echocardiograms only in the first 30 days of life were excluded from the analysis to mitigate the likelihood that pulmonary hypertension detected on the study was secondary to persistent pulmonary hypertension of the newborn or transitional physiology. In the final cohort, atrial septal defects were noted in 57 infants (17%), and 275 infants did not have ASD (82%). Of the infants with ASD, 15 (26%) had PH. In contrast, 34 (12%) infants without ASD had PH by echocardiogram.

The mean gestational age of infants with and without ASD was 26 weeks (± 2.2), and birth weight and birth length did not differ between groups (Table 1). Gender, self-reported race designation, and other antenatal characteristics such as betamethasone doses, intrauterine growth restriction, placental abruption, and chorioamnionitis also did not differ between groups. More mothers reported illicit drug use among the infants with ASD (20%), than those without ASD (9%). Infants with ASD also had slightly higher mean Apgar scores at 1 min of life (4.7 ± 2.6), compared to infants without ASD (3.9 ± 2.5, *p* = 0.031), although the Apgar scores at 5 min were similar between the groups. There was no difference in multiple gestation or mode of delivery between the groups. Hospital diagnoses, such as receipt of caffeine therapy, intraventricular hemorrhage, necrotizing enterocolitis, and retinopathy of prematurity were similar between ASD groups. There were no differences in the length of hospital stay, the number of positive blood, urine or cerebrospinal fluid infections, or surgeries between the categories. Over one-third of infants required some respiratory support at 28 days of life, but this proportion did not differ between groups. However, more infants with ASD had patent ductus arteriosus that were noted to be moderate or large in size (31.1 vs. 17.8%, *p* = 0.04). The mean number of echocardiograms were 2 in both groups, but a higher proportion of infants with ASD had PH by echocardiogram. Over double the proportion of infants with ASD (26.3%) had PH when compared with infants without ASD (12.3%, *p* = 0.006). The overall death rate for the cohort was 12.3%, and the proportion of infants who died and the timing of death did not differ between ASD categories.

**Table 1:** Associations between clinical covariates and ASD.

**Variable–Birth**	**Overall (*n* = 334)**	**ASD (*n* = 57)**	**No ASD (*n* = 275)**	***p*-value^a^**
Gestational age Mean (SD)	26.0 (2.2)	26.2 (2.2)	26.0 (2.2)	0.392
Birth weight (g) Mean (SD)	799 (238)	830 (236)	793 (238)	0.279
Birth length (*n* = 235) Mean (SD)	33.1 (4.0)	34.0 (3.5)	32.9 (4.1)	0.106
Gender (male) (*n* = 332) *n* (%)	197 (59.3%)	30 (52.6%)	167 (60.7%)	0.257
Maternal Race (*N* = 327) *n* (%)
African-American	209 (63.9%)	40 (70.2%)	169 (62.6%)	0.279
Non-African American	118 (36.1%)	17 (29.8%)	101 (37.4%)
Betamethasone (*N* = 287) *n* (%)			
2 or more doses	173 (60.3%)	29 (60.4%)	144 (60.3%)	0.983
Less than 2 doses	114 (39.7%)	19 (39.6%)	95 (39.8%)
Intrauterine growth restriction (*N* = 326) *n* (%)	30 (9.2%)	4 (7.4%)	26 (9.6%)	0.798
Placental abruption (*N* = 327) *n*, %	38 (11.6%)	6 (11.1%)	32 (11.7%)	0.898
Chorioamnionitis (*N* = 326) n (%)	24 (7.4%)	2 (3.7%)	22 (8.1%)	0.393
Illicit drug use (*N* = 295) *n* (%)	32 (10.9%)	10 (20.0%)	22 (9.0%)	0.022*
Drug use (*N* = 294) *n*, %	29 (9.9%)	8 (16.3%)	21 (8.6%)	0.114
Apgar 1 min (*N* = 325) Mean (SD)	4.0 (2.5)	4.7 (2.6)	3.9 (2.5)	0.031*
Apgar 5 minute (*N* = 325) Mean (SD)	6.5 (2.2)	7.0 (2.1)	6.5 (2.2)	0.122
Multiple gestation (*N* = 332) *n* (%)	55 (16.6%)	8 (14.0%)	47 (17.1%)	0.572
Delivery mode, Vaginal (*N* = 331) *n* (%)	112 (33.8%)	21 (37.5%)	91 (33.1%)	0.525
**Variable–Hospital**	**Overall**	**ASD**	**No ASD**	***p*****-value**^a^
Caffeine (*N* = 331) *n* (%)	206 (62.2%)	37 (67.3%)	169 (61.2%)	0.399
Intraventricular hemorrhage (y/n) (*N* = 331) *n* (%)	73 (22.1%)	13 (23.6%)	60 (21.7%)	0.757
Necrotizing enterocolitis (y/n) (*N* = 331) *n* (%)	84 (25.4%)	18 (32.7%)	66 (23.9%)	0.170
Retinopathy of prematurity (y/n) (*N* = 331) *n* (%)	184 (55.6%)	31 (56.4%)	153 (55.4%)	0.899
Length of stay Mean (SD)	80.7 (72.0)	78.9 (80.4)	81.1 (70.4)	0.838
Blood infection *n* (%)	46 (13.8%)	5 (8.8%)	41 (14.8%)	0.229
Urine infection *n* (%)	27 (8.1%)	4 (7.0%)	23 (8.3%)	1.000
Cerebrospinal fluid infection *n* (%)	5 (1.5%)	0 (0.0%)	5 (1.8%)	0.593
Surgery *n* (%)	301 (0.1%)	53 (93.0%)	248 (89.5%)	0.426
Respiratory support at 28 days (*N* = 332)	111 (33.4%)	21 (37.5%)	90 (32.6%)	0.479
Patent ductus arteriosus size (*n* = 253), *n* (%)
None/tiny/small	202 (79.8%)	31 (68.9%)	171 (82.2%)	0.043*
Mod/large	51 (20.2%)	14 (31.1%)	37 (17.8%)
Number of echocardiograms Mean (SD)	2 (1, 3)	2 (1, 3)	2 (1, 2.5)	0.061
Pulmonary hypertension *n* (%)	49 (14.7%)	15 (26.3%)	34 (12.3%)	0.006*
Death n (%)	41 (12.3%)	9 (15.8%)	32 (11.6%)	0.375
Time to death (*N* = 41) Median (IQR)	116 (56, 194)	96 (68, 208)	118 (50, 187)	0.581

*Infants with echocardiograms performed after 30 days of life were categorized as having atrial septal defect (ASD) if their study showed “ASD or ASD vs. patent foramen ovale.” Maternal race and other prenatal variables were abstracted from the infant medical record. Discrete hospital variables were abstracted from the clinical data warehouse, and echocardiographic data was manually abstracted as indicated in the Methods*.

a Chi-square or T-test performed, *Indicates statistical significance (p < 0.05).

When evaluated by outcome categories, infants with PH had a lower mean gestational age (25.2 ± 2 vs. 26.2 ± 2.2, *p* = 0.005), and a lower mean birth weight (699 ± 174 vs. 816 ± 243, *p* = 0.001), when compared with infants without PH (Table 2). There were no differences in other antenatal factors between infants with and without PH. Interestingly, a higher proportion of infants with PH had maternal African-American race than infants of mothers of other races (83.7 vs. 16.3%, *p* = 0.002). In perinatal history, the Apgar scores at 1 and 5 min were lower for infants with PH (2.9 ± 2.0 vs. 4.2 ± 2.5 at one minute, *p* = 0.002; 5.8 ± 2.0 vs. 6.5 ± 2.2 at five minutes, *p* = 0.011) than for infants without PH, but there was no difference in the mode of delivery between groups. Additionally, the number of infants with intrauterine growth restriction was not different between groups. The proportion of infants with necrotizing enterocolitis or surgery for necrotizing enterocolitis was not different between groups. However, a larger percentage of infants with retinopathy of prematurity (72.9 vs. 52.7%, *p* = 0.009) or intraventricular hemorrhage (41.7 vs. 18.7%, *p* < 0.001) also had PH, suggesting that the infants with PH were potentially a sicker group of infants throughout their neonatal course. In keeping with these data, infants in the PH group also had more positive urine cultures (16.3% vs. 6.7%, *p* = 0.041), and more positive tracheal cultures (38.5 vs. 23.2%, *p* = 0.020) than infants in the No PH group. Of note, 90% of the overall cohort had received some type of surgery, and 98% of infants in the PH group received surgery, indicating this was a high acuity, referral-based neonatal population. Of the infants with PH, 30.6% had at least one echocardiogram that showed ASD compared with 14.7% of the No PH group (*p* = 0.006). Although there were few differences in the proportions of infants with no ASD or only PFO between PH groups, a higher proportion of infants had PFO vs. ASD or ASD in the PH group than in the no PH group (8.2 vs. 4.9% and 22.5 vs. 4.9%, respectively; *p* = 0.043). Just over one-quarter (26.5%) of infants with PH received sildenafil, and a small proportion of infants without PH diagnosed at the referral center also received sildenafil (4.2%, *p* < 0.001). In reviewing the medical records of the infants without PH that received sildenafil, the majority of infants had been started on the medication at an outside hospital. Therefore, there was not echocardiographic documentation of pulmonary hypertension in our health system, and we chose not to reclassify these infants to the PH outcome category. Four percent of infants in the PH group were also treated with bosentan, whereas none of the infants in the No PH group were on bosentan. A small percentage of the infants had left ventricular dysfunction or hypertrophy, but these proportions did not differ by PH status. However, nearly one-quarter of infants with PH had right ventricular dysfunction (23.9 vs. 3.2%, *p* < 0.001), and over one-half of infants with PH had right ventricular dilation (52.1 vs. 8.6%, *p* < 0.001) or right ventricular hypertrophy (51.2 vs. 10.1%, *p* < 0.001). Nearly all of the infants with PH had evidence of moderate or severe septal flattening (97.7 vs. 52.1%, *p* < 0.001), and 40.8% also had a moderately or severely elevated tricuspid regurgitation gradient (40.8 vs. 0.0%, *p* < 0.001). The day of life of the echocardiogram that showed PH was a mean of 120.5 days (± 45.5) for infants in the PH group, and the last echocardiogram of the neonatal hospital course (for infants without PH) was 92.5 days (± 56.8, *p* = 0.001).

**Table 2:** Associations between clinical covariates and Late PH.

**Variable**	**Overall (*N* = 334)**	**Late PH (*N* = 49)**	**No PH (*N* = 285)**	***p*-value^a^**
Gestational age Mean (SD)	26.0 (2.2)	25.2 (2.0)	26.2 (2.2)	0.005*
Birth Weight (g) Mean (SD)	799 (238)	699 (174)	816 (243)	0.001*
Birth length (cm) (*n* = 235), Mean (SD)	33.1 (4.0)	32.2 (3.3)	33.2 (4.1)	0.199
Multiple gestation (*n* = 332) *n* (%)	55 (16.6%)	5 (10.4%)	50 (17.6%)	0.215
Betamethasone (*n* = 287) *n* (%)	173 (60.3%)	22 (55.0%)	151 (61.1%)	0.462
Drug use (*n* = 294) *n* (%)	29 (9.9%)	6 (14.3%)	23 (9.1%)	0.275
Illicit drug use (*n* = 295) *n* (%)	32 (10.9%)	8 (19.5%)	24 (9.5%)	0.062
Chorioamnionitis (*n* = 326) *n* (%)	24 (7.4%)	5 (11.1%)	19 (6.8%)	0.351
Placental abruption (*n* = 327) *n* (%)	38 (11.6%)	6 (13.0%)	32 (11.4%)	0.745
Intrauterine growth restriction (*N* = 326)	30 (9.2%)	5 (11.1%)	25 (8.9%)	0.584
Gender, male (*n* = 334)	197 (59.3%)	31 (63.3%)	166 (58.7%)	0.544
Maternal Race (*n* = 327)
African American	209 (63.9%)	41 (83.7%)	168 (60.4%)	0.002*
Non-African American	118 (36.1%)	8 (16.3%)	110 (39.6%)
Delivery mode (*n* = 331) *n* (%)				0.485
Vaginal	112 (33.8%)	18 (38.3%)	94 (33.1%)
C-section	219 (66.2%)	29 (61.7%)	190 (66.9%)
Apgar 1 Mean (SD)	4.0 (2.5)	2.9 (2.0)	4.2 (2.5)	0.002*
Apgar 5 Mean (SD)	6.5 (2.2)	5.8 (2.0)	6.5 (2.2)	0.011*
Caffeine therapy (*N* = 331) *n* (%)	206 (62.2%)	26 (54.2%)	180 (63.6%)	0.212
Necrotizing enterocolitis (*N* = 331) *n* (%)	84 (25.4%)	9 (18.8%)	75 (26.5%)	0.254
Retinopathy of prematurity (*N* = 331) *n* (%)	184 (55.6%)	35 (72.9%)	149 (52.7%)	0.009*
Intraventricular hemorrhage (*N* = 331) *n* (%)	73 (22.1%)	20 (41.7%)	53 (18.7%)	< 0.001*
Respiratory support at 28 days (*N* = 332) *n* (%)	111 (33.4%)	12 (25.5%)	99 (35.0%)	0.151
Blood infection *n* (%)	46 (13.8%)	10 (20.4%)	36 (12.6%)	0.145
Cerebrospinal fluid infection *n* (%)	5 (1.5%)	1 (2.0%)	4 (1.4%)	0.550
Urine infection, *n* (%)	27 (8.1%)	8 (16.3%)	19 (6.7%)	0.041*
Respiratory Infection, *n* (%)	85 (25.5%)	19 (38.8%)	66 (23.2%)	0.020*
Sepsis	67 (20.1%)	15 (30.6%)	52 (18.3%)	0.046*
Surgery, *n* (%)	301 (90.1%)	48 (98.0%)	253 (88.8%)	0.065
NEC surgery *n* (%)	73 (21.9%)	10 (20.4%)	63 (22.1%)	0.791
Ever ASD *n* (%)			
ASD, PFO vs. ASD	57 (17.1%)	15 (30.6%)	42 (14.7%)	0.006*
Other	277 (82.9%)	34 (69.4%)	243 (85.3%)
ASD Categories, *n* (%)			
None/trivial	44 (13.2%)	4 (8.2%)	40 (14.1%)	0.043*
PFO	232 (69.7%)	30 (61.2%)	202 (71.1%)
PFO vs. ASD	18 (5.4%)	4 (8.2%)	14 (4.9%)
ASD	39 (11.7%)	11 (22.5%)	28 (9.9%)
Sildenafil *n* (%)	25 (7.5%)	13 (26.5%)	12 (4.2%)	< 0.001*
Bosentan *n* (%)	2 (0.6%)	2 (4.1%)	0 (0.0%)	0.021*
Left ventricular dysfunction (y/n) (*N* = 329) *n* (%)	17 (5.2%)	3 (6.1%)	14 (5.0%)	0.727
Left ventricular hypertrophy (y/n) (*N* = 300) *n* (%)	12 (4.0%)	1 (2.2%)	11 (4.3%)	1.000
Ventricular septum intact (y/n) (*N* = 293) *n* (%)	16 (5.5%)	2 (4.4%)	14 (5.7%)	1.000
Right ventricular dysfunction (y/n) (*N* = 327) *n* (%)	20 (6.1%)	11 (23.9%)	9 (3.2%)	< 0.001*
Right ventricular dilation (y/n) *n* (%)	48 (15.0%)	25 (52.1%)	23 (8.6%)	< 0.001*
Right ventricular hypertrophy (y/n) (*N* = 319) *n* (%)	50 (15.7%)	22 (51.2%)	28 (10.1%)	< 0.001*
Septal flattening *n* (%)			
None/mild	71 (37.6%)	1 (2.3%)	70 (48.0%)	< 0.001*
Mod/severe	118 (62.4%)	42 (97.7%)	76 (52.1%)
Tricuspid regurgitation gradient (*N* = 330) *n* (%)			
None	286 (86.7%)	11 (22.5%)	275 (97.9%)	< 0.001*
Mild	24 (7.3%)	18 (36.7%)	6 (2.1%)
Moderate/severe	20 (6.1%)	20 (40.8%)	0 (0.0%)
SBP (*N* = 290) *n* (%)	78.2 (18.4)	80.0 (17.1)	77.9 (18.6)	0.475
DBP (*N* = 289) *n* (%)	44.1 (13.9)	43.5 (11.7)	44.2 (14.3)	0.764
Day of life, echocardiogram Mean (SD)	96.6 (56.1)	120.5 (45.5)	92.5 (56.8)	0.001*

*Infants with echocardiograms performed after 30 days of life were categorized as having Late PH or no Late PH. Echocardiograms were performed by clinical pediatric cardiologists and quantitative variables (tricuspid regurgitation gradient) and qualitative variables (shunt directions, septal flattening, degree of right ventricular dysfunction/dilation/hypertrophy) were measured. Systolic (SBP) and diastolic (DBP) blood pressure were measured at the time of the echocardiogram that showed PH. Sildenafil and bosentan use was extracted from the clinical data warehouse*.

a Chi-square or T-test performed, *Indicates statistical significance (p < 0.05).

**Figure 2:**
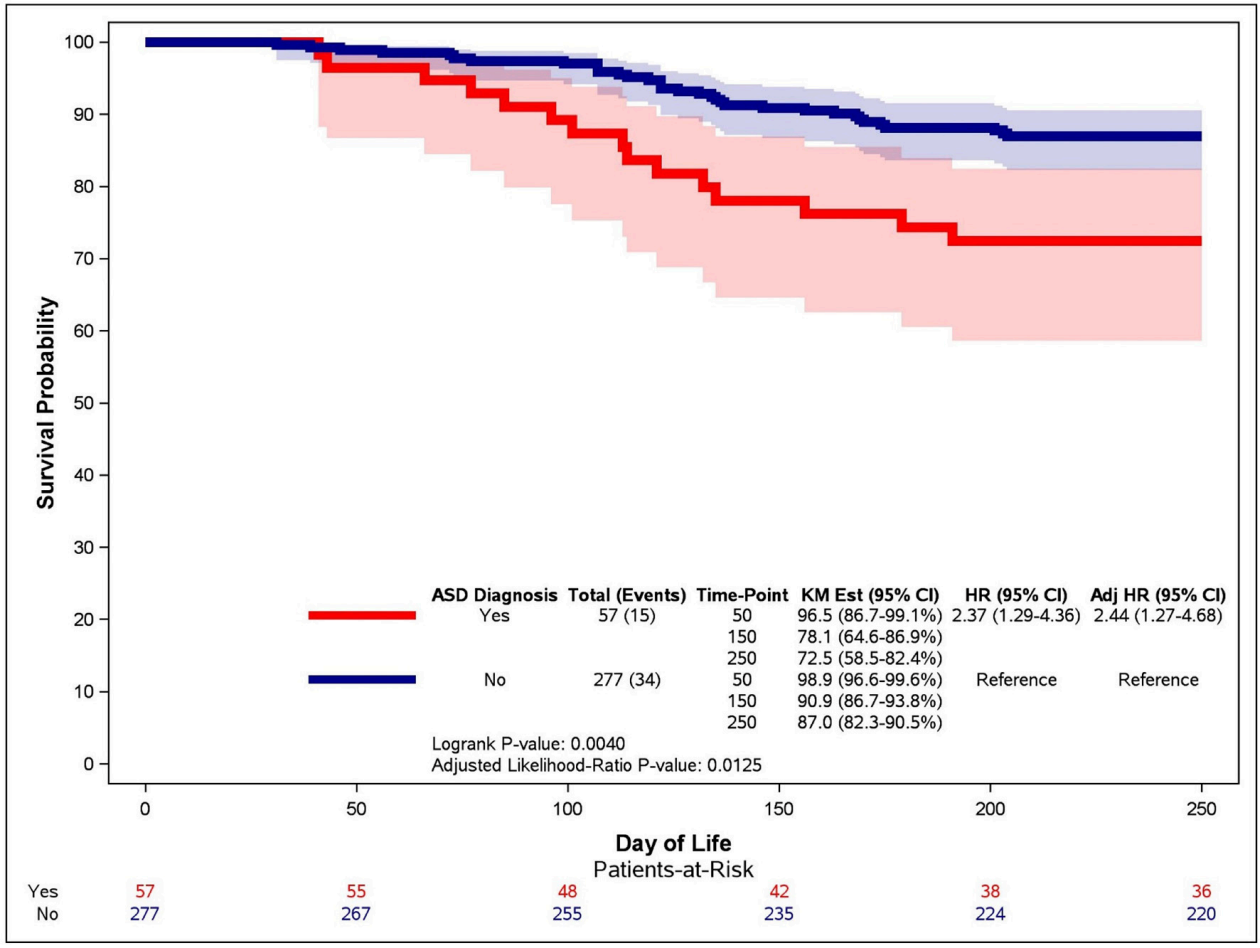
Kaplan-Meier curve for the association between ASD and time to PH. An unadjusted Kaplan-Meier curve was generated for infants with (red line) and without (blue line) atrial septal defects (ASD). Late PH was determined on echocardiographic studies performed after 30 days of life and infants were censored at death or 250 days of life.

When evaluating infants over time, neonates with an ASD had an unadjusted hazard ratio that was 2.37 (1.29–4.36) for the development of PH over infants without ASD (Figure 2, red line vs. blue line). When respiratory support requirement at 28 days, sepsis, African-American maternal race, birthweight and intrauterine growth restriction were controlled, the effect of ASD on the risk of PH development increased (HR 2.44, 95% CI 1.27–4.68). At day 50, the percentage of infants with ASD who did not have PH was 96.5% (CI 86.7–99.1%), slightly lower than that of infants without ASD (98.9%, CI 96.6–99.6%). By day 150, the difference in the probability of survival without PH between infants with and without ASD had widened. Approximately nine out of ten infants without ASD (90.9%, CI 86.7–93.8%) survived without PH, and almost one out of ten had PH. In contrast, only 8 out of 10 infants with ASD survived without PH, and nearly one in five developed PH (78.1%, CI 64.5–86.9%). This difference was moderately increased beyond 150 days, with 87% of infants without ASD (CI 82.3–90.5%) and 72.5% infants with ASD (CI 58.5–82.4%) with survival free of PH.

Utilizing significant clinical variables that were associated with the PH outcome in our descriptive analyses, and forcing in factors that are reported in the literature to be associated with PH, such as birthweight, intrauterine growth restriction, and respiratory support requirements, we developed a multivariable Cox proportional hazard model for the development of PH (9). After controlling birth weight, intrauterine growth restriction, maternal race, sepsis, and respiratory support at 28 days, infants who had ASD had a hazard of PH development of 2.44 (95% CI 1.27–4.68) over infants without ASD (Table 3). Respiratory support and intrauterine growth restriction were not significantly associated with PH when other factors were held constant. However, infants with “sepsis,” defined as having a positive blood culture in the microbiology laboratory, had a 2.31 increased hazard for late PH (95% CI 1.19–4.49). Maternal African-American race also had an increased hazard of PH over infants of other races (White, Asian or Hispanic) (HR 3.34, 95% CI 1.39–8.06). In keeping with other studies, infants who had a birth weight below 800 grams had a greater hazard for the development of late PH than those of higher birth weight (HR 2.24, 95% CI 1.09−4.59).

**Table 3:** Cox proportional hazard model for the time to late PH.

**Variable**	**Unadjusted**		**Adjusted**	
	**HR (95% CI)**	***p*-value**	**HR (95% CI)**	***p*-value**
Atrial septal defect (Yes vs. No)	2.37 (1.29, 4.36)	0.005*	2.44 (1.27, 4.68)	0.007*
Respiratory support at 28 days(yes vs. no)	0.68 (0.35, 1.29)	0.237	0.73 (0.36, 1.46)	0.366
Sepsis (yes vs. no)	1.76 (0.96, 3.22)	0.070	2.31 (1.19, 4.49)	0.013*
African American Maternal race (yes vs. no)	3.00 (1.41, 6.40)	0.005*	3.34 (1.39, 8.06)	0.007*
Birthweight (< 800 g vs. >800 g)	2.66 (1.36, 5.20)	0.004*	2.24 (1.09, 4.59)	0.028*
Intrauterine growth restriction (yes vs. no)	1.36 (0.54, 3.45)	0.541	1.63 (0.63, 4.23)	0.319

*The hazard for the development of late pulmonary hypertension was determined using unadjusted Kaplan-Meier survival probabilities. In adjusted Cox proportional hazard models, the effect of atrial septal defect on the outcome of late PH was determined after controlling for variables that were significant in descriptive analyses (sepsis, African American maternal race, and birth weight) or in the literature (respiratory support, intrauterine growth restriction). Hazard ratios (HR) and 95% confidence intervals (CI) were determined. Statistical significance was defined as p-value < 0.05*.

## Discussion

In this study, we have found that very low birthweight infants with an ASD are over two-fold more likely to develop late PH in the first 250 days of life than infants without an ASD. In the first 50 days of life, the difference in hazard for PH between infants with ASD and without ASD is minimal. However, by 150 days of life, 22% of infants with ASD have developed PH compared with only 10% of infants without ASD, and the hazard for PH in these groups remains divergent throughout our study. Given that the mean gestational age at birth for infants with or without ASD was 26 weeks, the time period associated with the greatest risk of PH diagnosis in our cohort was between 33 and 47 weeks. Beyond 150 days of life (or approximately 47 weeks), a much smaller percentage of at-risk infants either with or without ASD were newly diagnosed with PH. These data suggest that echocardiographic screening efforts for premature infants who are at risk of PH should be initiated in the late preterm period, when infants are approximately 32–33 weeks, and continue until at least a few months post-term. Most PH in our cohort was diagnosed by approximately 48 weeks, and this finding is in keeping with other reports that have found the majority of PH to be diagnosed by 2 to 5 months postnatal age (6, 23, 27). Given that neonates who are born at less than 32 weeks gestation have over a 3-fold increase in late PH, even after adjustment for other significant clinical variables, echocardiographic screening strategies should potentially be targeted to this high-risk period for most premature infants with BPD (11, 14).

The contribution of left-to-right shunts in the development of PH has been suggested in other studies. However, no other studies have evaluated the effect of ASD on the timing of PH diagnosis. In one group of high-risk infants who were being followed in a BPD clinic, premature neonates who required ligation of a patent ductus arteriosus during their hospital course were over twice as likely to have PH at 2 months of life (28). Further, very low birth weight infants with ASD at 2 months of age had a three-fold higher odds of PH diagnosis than those without ASD (23). In a separate series of infants being followed in a referral clinic, more infants with severe PH had a secundum ASD on echocardiography than infants with less-than-severe PH (< 50% systemic pressure) (6). However, in this same study, 3 out of 4 infants with ASD and PH had normalization of pulmonary vascular resistance and improved pulmonary blood flow in response to oxygen and inhaled nitric oxide, suggesting a reactivity to the vasculature of infants with left-to-right shunts that may benefit from earlier identification. Optimal timing of ASD closure is problematic given that some proportion of left-to-right shunts close spontaneously (29). In infants in whom ASDs persist, closure of the defect may improve respiratory and PH outcomes. In a small study of infants with BPD with secundum ASDs, 11 out of 13 had improved respiratory outcome and 4 out of 13 had improvement in PH following closure before 12 months of age (26). In other series, approximately half of infants with PH had improvement in their pulmonary vascular pressures following shunt closure (17, 18, 29). Although a large majority of ASDs that are < 3 mm in diameter spontenously close, moderate, or larger defects can grow over the first few years of life and become hemodynamically more significant (30). As such, premature infants with ASD may warrant closer follow-up to ensure that left-to-right shunting is not contributing to pulmonary vascular resistance changes and early closure is not indicated.

Our findings that infants with birthweight less than 800 grams had a much higher odds of PH, even with other clinical factors controlled, is congruent with the findings of other literature (1, 9, 13). Although we did not find differences in the degree of respiratory support at 28 days, it is possible that some of the increased risk for PH by birthweight is a reflection of the higher incidence of BPD at 36 weeks corrected gestational age and altered vascular growth in more premature infants. Additionally, maternal self-reported African-American race was strongly associated with PH in our study, even after other significant clinical variables including intrauterine growth restriction were controlled. Adult studies are beginning to appreciate differential effects of hypoxia tolerance, cardiac physiology, and even mortality associated with PH by ethnicity, but the contribution of these personalized factors to PH in premature infants has not been fully examined (31). In our study, because of the large proportion of African-American mothers relative to other races, we grouped race dichotomously, but further, more thorough, evaluations of PH risk based on ethnicity should now be considered.

We recognize that our study has limitations due to its retrospective nature, and the nature of its referral, neonatal population. The neonates in our cohort were admitted to neonatal intensive care units in quaternary, referral care centers, and, as such, may not be representative of healthier premature infants. However, the Kaplan-Meier and Cox proportional hazard analyses allowed us to evaluate a dynamic population of infants with ASD or no ASD with respect to the timing of PH diagnosis the first 250 days of life, providing information that may be helpful to guide bedside clinicians in determining who to focus screening efforts on, and when to optimally order echocardiographic studies. Additionally, infants categorized with ASD in our cohort had a diagnosis of either “ASD” or “PFO vs. ASD,” and infants categorized with no ASD had either an isolated “PFO” or no atrial level communication, by echocardiographic report. In general, at our center, a diagnosis of ASD or PFO vs. ASD suggests an atrial communication that is larger than 3 mm in diameter with an atrial level shunt. However, we recognize that it is a limitation of our study that we did not utilize a research protocol to specifically define the size and shunting necessary for each level of ASD, and that using a clinical definition may have led to some misclassification in the exposure groups. Further, we recognize that echocardiographs were ordered at clinical discretion, and as such, there may be ascertainment bias in certain groups over others. In fact, infants with PH appeared to be a sicker group, with higher rates of retinopathy of prematurity, intraventricular hemorrhage, and sepsis, and lower birthweight compared with infants without PH. In spite of this, when variables associated with clinical acuity, such as birthweight, respiratory support, sepsis, and intrauterine growth restriction were held constant, ASD continued to double the odds of PH diagnosis in our population. Finally, we recognize that some of the measures used to echocardiographically detect PH, namely septal flattening and tricuspid regurgitant jet velocity, can be altered by the presence of a left-to-right shunt even when pulmonary vascular resistance is not elevated and that definitive PH diagnosis would require cardiac catheterization. Further, tricuspid regurgitant jet velocity can be detected in only 15–61% of echocardiographic studies, and the positive predictive value of echocardiography for PH in the absence of a tricuspid regurgitant jet is limited to between 50 and 83% (4, 32, 33). In spite of these limitations, fewer than 10% of infants have cardiac catheterization performed, and echocardiography remains the primary modality to diagnose PH in hospitalized neonates (29, 32). Over half of our PH cohort had right ventricular dilation or hypertrophy (RVD Late PH 52.1 vs. 8.6% No PH; RVH Late PH 51.2 vs. 10.1% No PH), and almost one-quarter had right ventricular dysfuction (Late PH 23.9 vs. 3.2% No PH) indirectly suggesting that the majority of the infants in our PH category were true positives, and not infants exhibiting septal flattening as a result of increased left-to-right flow.

Overall, this study shows that infants with ASDs have over double the odds of PH, even when clinically significant variables are controlled. Further, it demonstrates that the majority of PH is diagnosed for premature infants in both ASD categories by 150 days of life, but more infants with ASD may be diagnosed at >150 days than infants without ASD. Lastly, it suggests that specific ethnic groups, such as African-Americans, may have a higher odds of PH, although this association should be further evaluated in a larger and more racially comprehensive study cohort. In conclusion, our investigation provides evidence that premature infants, in a referral neonatal intensive care unit, who have an ASD are at particular risk for PH development, and that echocardiographic screening efforts should be continued for at least a few months of postnatal age for these high-risk infants. Prospective research studies that evaluate echocardiographic findings in a protocolized manner may want to target evaluations to this population within the first several months of postnatal life.

## Author contributions

SV-R, CT, UK: Study design; SV-R, CT, PS: Data acquisition and analysis; SV-R, CT: Figure preparation; SV-R, LG, UK: Manuscript preparation; SV-R, LG, UK: Manuscript editing; SV-R, LG, UK: Scientific guidance and oversight.

### Conflict of interest statement

The authors declare that the research was conducted in the absence of any commercial or financial relationships that could be construed as a potential conflict of interest.
